# Eye Movements during Art Appreciation by Students Taking a Photo Creation Course

**DOI:** 10.3389/fpsyg.2016.01074

**Published:** 2016-07-14

**Authors:** Chiaki Ishiguro, Kazuhiko Yokosawa, Takeshi Okada

**Affiliations:** ^1^Department of Educational Psychology, The University of TokyoTokyo, Japan; ^2^Department of Psychology, The University of TokyoTokyo, Japan; ^3^Interfaculty Initiative in Information Studies, The University of TokyoTokyo, Japan

**Keywords:** art appreciation, eye movements, art education, photography, saccades

## Abstract

Previous studies have focused on the differences in the art appreciation process between individuals, and indicated that novice viewers of artworks, in comparison to experts, rarely consider the creation process of the artwork or how this may relate to style. However, behavioral changes in individuals after educational interventions have not been examined. Art education researchers claim that technical knowledge and creation experiences help novice viewers to pay attention to technical features of artwork. Therefore, an artistic photo creation course was designed and conducted to help students acquire techniques and procedural knowledge of photo creation. The present study verified whether students' viewing strategies during appreciation of photographs changed after the course. Twenty-one students participated in two sessions, viewing the same 12 photographs before and after the course. Based on the analysis of recorded eye movements, the results indicated that the students' perceptual exploration became more active with photographs containing recognizable subjects (i.e., humans and objects), and their global saccades increased when they viewed classic photography, one of the categories of photography covered in the course. Interview data after the course indicated that students became aware of the technical effects in photographs. These results suggest that students' viewing strategies may change following a course, as assessed by behavioral measures of eye movements. Further examination is needed to validate this approach to educational effect measurement.

## Introduction

Does art appreciation change in individuals as a result of educational interventions, and how does it change? This is one of the critical questions in art education. Formal and informal educational institutes, such as schools and museums, provide educational programs and courses, offering laypeople, untrained in art, opportunities to improve their appreciation of it. When evaluating these educational interventions, art educators and researchers have typically focused on subjective, interpretative changes, as described in questionnaires and comment sheets, to analyze them qualitatively (e.g., Housen, 1987; Parsons, 1987).

A recent study conceptualized asthetic appreciation as complex and sequential information processing. Leder et al. (2004) proposed the information-processing stage model of asthetic appreciation, which involved five stages: perception, explicit classification, implicit classification, cognitive mastering and evaluation. The revised version of the model (Leder and Nadal, 2014) claimed that there was an automatic process, which included perception, explicit classification, implicit classification, and a deliberate process consisting of cognitive mastering and evaluation. In addition, it suggested that the automatic process and the cognitive interpretation process interact with each other in art appreciation. In other words, the interpretation and evaluation are affected by a primary process of appreciation, including perception, explicit classification and implicit classification, and vice versa. Therefore, when researchers study changes in the individual appreciation process, it is significant to focus on the process of appreciation, not only its result.

If there were a reliable method to measure the process of appreciation, the evaluation of the educational effects of such courses would be advanced. As an initial step in this direction, this study examines whether the process of appreciation in laypeople, untrained in art, changes after educational interventions, and if so, what kind of change there is.

### Difference in photo viewing process with the application of knowledge

People view images mainly for two purposes: to recognize objects and models, and to appreciate them asthetically. The difference in the viewing process of images between individuals is especially prominent in the case of photography, which was invented as a means to produce images but has since been transformed into a type of fine art. Although, people can view photos simply for recognition, which depends purely on the automatic process described in the model of Leder et al. (2004), asthetic appreciation depends on a person's specific knowledge and experience of artistic photography and photo creation, which corresponds to the deliberate process in Leder's model.

Appreciation of photography is influenced by knowledge of two areas: art history and creation. Critics and photographers who are experts appreciate fine art photography by referring to both areas of knowledge. Critics focus on the evaluation of artwork. They enjoy the asthetic features and visualize the creation process by applying knowledge of art history. They then interpret it from their perspective and evaluate it. Photographers appreciate artwork by applying knowledge and experience of photo creation, while they also evaluate the work of others as critics do. For example, when they view a photo of beautiful scenery, they may take pleasure from viewing the photo, or discover a new technique from the photo and try to imagine what the creator wanted to show. If they appreciate a new technique in a photo, they may sometimes experiment with it in their own artistic creations or modify it for their own purposes. Thus, photographers interpret the creation process of the photo and if possible apply it to their own creation process.

These two types of appreciation in critics and photographers occur commonly as they visualize the creation process, although the purposes and benefits of appreciation may differ between them. Bullot and Reber (2013) argued that sensitivity to art-historical contexts is connected to the process of art appreciation, and visualizing the creation process of artwork is one of the requirements for artistic understanding. Therefore, interpreting the creation process behind artwork seems to be one of the essential processes driving art appreciation in experts.

However, compared to experts, novices rarely focus on the creation process, but focus instead on the objects and models in the photo. This difference between novices and experts has been described in studies on the process of art appreciation. Specifically, studies suggest that novices rarely pay attention to styles and visual features of artwork, but instead focus on the contents depicted in the work (e.g., Parsons, 1987; Winston and Cupchik, 1992). This type of processing in appreciation does not entail visualizing the creation process. However, if novices can increase their knowledge and experience of art, they may apply this in their appreciation, particularly when they interpret the creation process.

### Effective educational interventions to improve photo appreciation

Formal and informal educational institutes, such as universities and museums, provide educational programs, and courses in art creation. These educational interventions help novices gain knowledge and experience of creation. As a result, novices may visualize the creation process using their knowledge of artistic techniques and expressions. Thus, if novices can acquire knowledge and experience of art creation through educational interventions relating to photography, they can develop their level of expertise and ability to interpret the creation process of photography.

What kind of educational interventions are effective in improving novices' appreciation of photography? The purpose of art appreciation education has thus far been to promote various interpretations of artwork by students. For this purpose, educational programs for art appreciation have used methods to provide a variety of information about artwork. For example, in the classroom, teachers provide students with art history lectures and explanations about works of art. In museums, some curators help viewers interpret artworks by discussing them through lectures and workshops (Dialogical Appreciation; Arenas, 2001). Other curators guide viewers in interpreting artwork from various points of view according to viewers' cognitive development (Visual Thinking Strategy; Housen, 1987). These educational methods of appreciation attempt to promote learners' varied interpretation of artworks by providing information relating to the artworks and integrating it through discussions and dialogues. Certainly, such practices are useful for learners when they integrate their interpretation with visual information. However, if educators focus on prompting learners to understand the creation process of artwork, it may be necessary to provide more specific knowledge about art creation through educational interventions.

It has been suggested that both of art historical knowledge and techniques of creation play important roles in art appreciation (Eisner, 1972; Wilson and Wilson, 1987). In addition, it has been suggested that style-related cognitive processes influence artistic interpretation (Belke et al., 2006). In order to promote artistic understanding, educators should provide learners with such knowledge. If a viewer acquires procedural knowledge for creation, their art-related interpretation and style-related processing will improve. Such changes may be reflected in the viewer's perceptual analysis (Leder et al., 2004).

Hence, this study proposes that educators should help learners acquire procedural knowledge and techniques for creation through technical instruction and training, in addition to providing declarative information about art history and works of art. For learners to acquire procedural and technical knowledge, several lectures and training sessions are required, whereas declarative knowledge can be acquired in a single session of instruction. Therefore, this study was conducted over a long-term (3-month) course.

### Measuring perceptual processes in art appreciation

If learners acquire procedural and technical knowledge of creation through an art course and their viewing processes change, how can these changes be measured? To date, learners' appreciation has been assessed through verbal protocols in their comments and writing in art appreciation studies. These studies argue that the content of verbal protocols develops with artistic experience (Housen, 1987; Parsons, 1987). However, on the basis of verbal descriptions alone, it is difficult to determine whether learners just use their acquired knowledge in their verbal and written communications, or whether their way of viewing actually changes.

It may be suggested that eye movement is an alternative measure of viewing. Eye movement is recognized as a parameter that reflects visual information processing in the brain (Carpenter, 1977/1988). It is applied to research areas examining complex types of human intellectual processing, such as reading and problem solving. For example, the results of studies of problem solving that assess the process by eye movement suggest that learners' eye movement data provide indications of specific mechanisms of representational change (e.g., Knoblich et al., 2001; Grant and Spivey, 2003). These studies approach complicated information processing by applying eye movement measures. Extending this approach to photo viewing, researchers adapt the measures to assess viewing strategies in art appreciation.

It is considered that eye movement data include measures showing visual processing: fixation and saccades. Fixation is a measure that shows the viewer's focus. A saccade is a rapid eye movement between fixation points, which is used as a measure to show the order of visual processing and searching behavior. These are popular measures to examine differences in viewing strategies between individuals in art appreciation (Nodine et al., 1993; Zangemeister et al., 1995; Vogt and Magnussen, 2005, 2007). Although they do not reflect the specific contents of viewers' interpretations, they do indicate where and how much viewers focus on the artwork, and how they explore the features in the artwork. Therefore, the present study uses eye movement data, specifically fixation and saccades, in order to examine whether learners' viewing strategies in art appreciation changed after an educational intervention.

### Research question

Do learners change their viewing strategies in art appreciation after a course of education, and if so, what kind of changes in viewing strategies do they demonstrate after the course?

One possibility is that learners' appreciation process changes to become more like that of artistically trained viewers. Based on Berlyne's theory of psychoaesthetics (Berlyne, 1971), perceptual exploratory behaviors in asthetic viewing can be categorized as diverse and specific. Although, such exploratory behaviors have not been examined statistically, Nodine et al. (1993) operationalized them by duration of fixation, and concluded that the difference in exploratory patterns was affected by compositional factors in the images viewed. Other studies have shown that viewers with significant knowledge and experience of artistic creation have different scanning strategies in art appreciation. Zangemeister et al. (1995) examined scanpaths, the repetitive sequences of saccadic eye movements, and found that in viewing abstract paintings, artists and sophisticated viewers performed more global scanning than novices. Vogt and Magnussen (2007) confirmed that the ratio of global and local saccades differed in artistically untrained viewers and artists when viewing abstract pictures, including ones containing no recognizable features. Another difference resulting from the level of expertise is that untrained viewers pay greater attention than artistically trained viewers to pictorial elements, such as humans and objects, rather than visual composition. Nodine et al. (1993) indicated that artistically trained people show more interest in the relationship between objects than in individual objects in paintings. Vogt and Magnussen (2007) also showed that the viewing time of regions surrounding recognizable objects was higher in artistically trained viewers than viewers without artistic training.

Hence, artistically trained viewers demonstrate more diverse exploration and global saccades when viewing abstract pictures, and show less interest than non-artistically trained viewers in recognizable features, such as humans and objects in realistic pictures. If these characteristics of the appreciation process are affected by the accumulation of knowledge and experience of artistic creation, the appreciation process of learners may become similar to artistically trained viewers after a course of instruction. Furthermore, the change may be affected by whether the pictures include recognizable features or not. In other words, learners' global saccades will increase when viewing photos that do not include recognizable subjects, and their interest in recognizable features when viewing photos with recognizable subjects will decrease after participating in an educational course (Hypothesis 1).

The changes in the appreciation process predicted in Hypothesis 1, above, may depend on the contents of educational interventions. Vogt and Magnussen (2007) proposed that the appreciation process of artistically trained viewers is the result of decades of learning experience in art. Such experience provides experts with various kinds of procedural knowledge and techniques for creation. Experts may interpret artistic techniques by applying specific knowledge to each work of art. Also, some theories on art education claim that artistic theory and technical knowledge promote a focus on the areas of an artwork that display artistic techniques (Eisner, 1972; Wilson and Wilson, 1987). Therefore, if learners acquire specific knowledge and techniques on a course, the characteristics of the appreciation process, such as their perceptual exploration, global saccades and interest in recognizable subjects, will change with the type of photographs that are covered in the course. In contrast, such changes will not be found with photographs that are not discussed on the course (Hypothesis 2).

To test these hypotheses, a fine art photography course was conducted for this study. It was designed to help learners acquire procedural knowledge and techniques for art creation, as well as providing declarative information about art history and works of art. Experiments were also conducted before and after the course (pre- and post-tests). In each experiment, the participants studied photographs with recognizable subjects and ones with unrecognizable subjects. Some types of photographs were dealt with in the course, and other types of photographs were not. The present study examined whether the participants' duration of fixation, frequency of global saccades and viewing time of recognizable subjects changed. It also examined whether such changes appeared with photographs containing recognizable subjects or unrecognizable subjects, and with which types of photography (covered in the course, or not). In interviews conducted after the tests, the participants answered questions about whether their appreciation had changed and how. Finally, the interview data was used to discuss whether learners' appreciation changed or not through the educational interventions, and how the interventions affected their appreciation of art.

## Methods

### Educational interventions in photo creation

**Figure 1:**
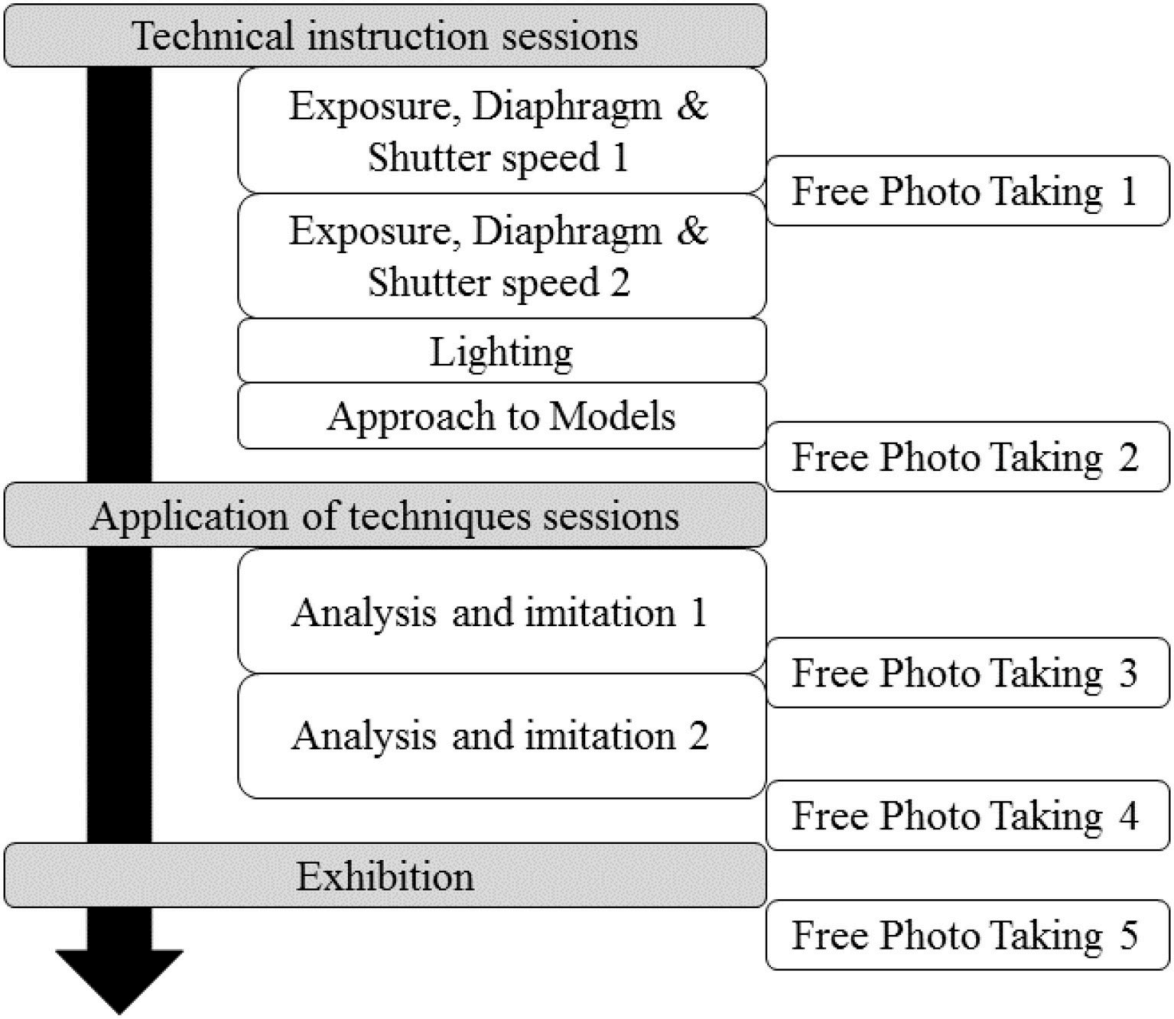
**The design of educational interventions in the artistic creation course**.

“Artistic Creation,” a fine art photography course for undergraduate students, was held at the University of Tokyo in the spring of 2012. The instructor was a photographer who teaches at a professional school of photography, and holds domestic and international exhibitions. He assisted students in acquiring procedural knowledge and techniques for photo creation. The first and third authors attended the course and provided guidance on academic assessment. The course consisted of 14 classes in total (see Table 1, Figure 1). Twenty-one undergraduates (10 males and 11 females) at the University of Tokyo in Japan participated in the course. Their ages varied from 20 to 27 (*M* = 21.33, *SD* = 1.58). None of them had previously received any professional education in artistic creation, including artistic photo creation. The students borrowed a digital single-lens reflex camera (DSLR) for each class, and some students used the camera outside the class for creation and practice. Five students used their own DSLR's. The students were informed that the course and their homework would be recorded and analyzed for this research.

**Table 1:** **The course schedule**.

1st class	Guidance
2nd class	Technical instruction class 1
3rd class	Free photo taking 1
4th class	Technical instruction class 2
5th class	Technical instruction class 3
6th class	Technical instruction class 4
7th class	Free photo taking 2
8th class	Application of techniques class 1
9th class	Free photo taking 3
10th class	Application of techniques class 2
11th class	Free photo taking 4
12th class	Exhibition
13th class	Free photo taking 5
14th class	Introduction to teacher's artworks

#### Course program

The artistic creation course included technical instruction sessions for acquiring basic technical knowledge and experience, and sessions on the application of techniques to train their eyes in order to appreciate photography as art (see Table 1, Figure 1).

There were four classes of technical instruction for the first intervention on the artistic creation course. The classes included instruction and practical training in basic technical and expression methods: exposure, diaphragm, shutter speed, lighting, and approach to models. These are fundamental techniques and expression methods for creating artistic photographs and understanding the procedure.

Two analysis and imitation classes were held in the sessions on the application of techniques after the technical instruction sessions. In the classes, the students examined an artistic photograph individually for several minutes. Then they shared and discussed their comments with each other in class. Once the discussion was finished, the class instructor provided the students with explanations about the photograph, its creator, and the characteristics of its creation and background. Next, the students imitated the technique of the photograph in order to learn the expression method that was used. The example used in the first analysis and imitation session was “Farm Girl” in “People of the twentieth Century” by August Sander (1874–1964), which is a collection of portrait photographs of people in the early twentieth century. The photograph used in the second session was “Los Angeles, California” by Garry Winogrand (1928–1984), which is a street photograph of people on a city street, and demonstrates the social problems hidden in everyday life. These two classes were designed to give instruction on two types of photographs: classic and street photographs. The instructor explained that the former type consisted of photographs taken using camera techniques in a studio setting, while the latter consisted of photographs of subjects in natural situations, without the use of certain camera techniques.

Apart from the technical instruction sessions and the application sessions, the course included classes in freestyle photography, and an exhibition. The students created their photo artwork in the classes on freestyle photography. They also selected five photographs that they had taken during the course as their artwork, showed them to the other students and the instructor, and received comments in the exhibition class. These classes did not support the students' appreciation directly; they were, however, designed to provide the students with opportunities to apply the knowledge and techniques learned in the technical instruction sessions, and the application sessions.

The students were given two kinds of homework in order to encourage reflection on their creations after each class. The first involved the task of explaining the photographs they had taken at the classes. The second kind entailed describing what they had considered and noticed about artistic creation each week, both inside and outside of the classes.

### Experiment

The present study conducted a quasi-experiment with a three-factorial design. The independent variables were session (pre- and post-test), recognizable subjects in photographs (with recognizable subjects and with unrecognizable subjects), and category of photography (control, classic, and street photography; the latter two were covered in the course). The dependent variables were measures from eye movements: average duration of fixation, frequency of saccades and time viewing the areas including recognizable subjects. All 21 students on the artistic creation course participated in the pre- and post-tests.

#### Artistic photographs for the experiments

The 12 photographs that were shown in the experiments were selected according to the hypotheses of this study. They were taken from a photography book (Koch, 2009) and divided into three types of photography, two of which were discussed on the course. Control photographs included depictions of still life and crowds. Photographs of this kind were not covered in the educational intervention. Classic and street photographs were photographs of humans and scenery. Photographs of these kinds were covered in the technical instruction and application sessions. Specifically, the classic photographs were of a type that used methods of expression that were taught in the technical instruction sessions and used in analysis and imitation session 1. The street photographs were of a type that used methods of expression that were taught in the technical instruction sessions and used in analysis and imitation session 2. The three types of photography were further divided into two groups: photographs with recognizable subjects and photographs with unrecognizable subjects. These classifications and a list of basic information about the photographs are shown in Table 2.

**Table 2:** **Classification of photographs used in the experiments**.

	**With recognizable subjects**			**With unrecognizable subjects**		
	**Artist**	**Year**	**Title**	**Artist**	**Year**	**Title**
Control (crowds and still life)	Andre Kertesz	1928	Fork	Tina Modotti	1926	Campesinos
	Edward Weston	1930	Pepper No. 30	Abbas	1989	Students of the Al Azhar college attend Friday prayer in the auditorium, transformed into a mosque for the occasion, Jakarta
Classic photography (portrait and scenery)	Bruce Davidson	1966	East 100th Street, New York City	Ansel Adams	1945	Mount Williamson, Sierra Nevada from Manzanar, California
	Guido Harari	2002	Lou Reed and Laurie Anderson	Gabriele Basilico	1991	Beirut
Street photography (snapshots)	Robert Doisneau	1957	Les Enfants de la Place Hebert	Sebastiao Salgado	1991	Working on an oil wellhead, Greater Burhan, Kuwait
	Dennis Stock	1955	James Dean, New York City	Raghu Rai	1972	Traffic at Chawri Bazaar crossing, Delhi

In addition, the photographs were selected according to whether they were familiar, attractive, and interesting to viewers or not, because familiarity, liking and interest in pictures are indicated to influence the viewing of art (Brieber et al., 2014). Ten graduate students (five males and five females, aged 22–28) of psychology at the University rated the 12 photographs on the three aspects on a 7-point Likert scale. Among the photographs selected, there were none for which any of the mean ratings were less than 2 or more than 6.

#### Apparatus

Eye movements were measured by eye tracking equipment, EMR-AT-VOXER (NAC Image Technology Inc., Aoyama, Tokyo). This consisted of an infrared camera located on a table directly in front of a 24.1-inch monitor showing 12 pictures successively at a screen resolution of 1600 × 1200 pixels. The size of the photographs ranged from 484 to 831 KB, and the width and length of the photographs were 666–1012 pixels, and 648–992 pixels, respectively. The camera recorded the participant's dominant eye and its eye movements. The eye movements were recorded by placing the participant's chin on the chin rest at a distance of 60 cm from the screen with pictures subtending visual angles of 11.7⋅ vertical, 16.1⋅ horizontal. Spatial and temporal resolutions were 0.3⋅ and 60 Hz, respectively.

Data was recorded on MiniDV and was analyzed with EMR-dFactory (NAC Image Technology Inc., Aoyama, Tokyo). Data output recorded vertical and horizontal eye movements, fixation, and duration of fixation. Fixation time was set at a minimum of 150 ms in the area within 100 pixels. Saccades were computed from the data files.

#### Procedure

The participants viewed three sets of four photographs (in total 12) both before (pre-test) and after (post-test) the course. On entering the laboratory, the experimenter explained the experiment, which was approved by Life Science Ethics and Safety, the Ethics Committee of the University of Tokyo (14–7), and all the participants gave permission for their eye information and interview data to be used in the study. The participants were then seated comfortably in front of a monitor and positioned at a chin rest. A calibration procedure was conducted with a nine-point grid stimulus. Then the participants were instructed to “examine each photograph freely.” They viewed six pictures in each of two sessions, with a few minutes rest between the sessions. Before the second session, the participants were again calibrated, and then instructed to view the other six photographs in the same way as in the first session. The pictures were shown in random order for each participant. The viewing time was 50 s for each picture, with a 20 s interval between each picture, during which the participants could close their eyes in order to minimize eyestrain. After the post-test, the participants were interviewed about the difference in viewing photographs between the beginning and the end of the course. In the interview, they answered the following questions: “Did your way of appreciation change after the course? If it changed, could you tell us about the specific change(s)?” The analysis counted the number of participants who answered “yes”, and then described their specific change or changes.

### Participants and measures for analysis

Although, all the students on the course participated in the pre- and post-tests, part of the data was missed in performing the experiments and in the process of data recording. The data of two participants in the post-test could not be recorded to MiniDV due to an operational failure. In addition, part of the eye movement data in some participants could not be detected accurately because of the reflection of some participants' contact lenses, and because of the misdetection of their eyebrows and eyeglasses. These operational failures and accidents in recordings could not be rectified by adding participants because of the limited number of participants on the course. Therefore, the researchers adopted the criterion for the data selection as whether more than 70% of the data pertaining to the participants in each session and for each photograph were recorded successfully or not. As a result, data from 13 of the participants was used for the subsequent analysis.

It has previously been shown (Antes, 1974) that visual processing for preference and specific analysis of pictures occurs within approximately 20 s of the beginning of the art appreciation. Therefore, the analyses of this study focused on data at 16 s after the beginning of each photo viewing session in order to examine the early phase of visual processing.

The eye movement measures were operationalized as follows. We computed the average duration of fixation to examine whether students' perceptual exploration changed from being diverse or specific. The frequency of saccades was calculated according to the definition as the movement from one fixation point to another with a velocity greater than 100⋅/s (Tursky, 1974; Osaka et al., 1993). In addition, saccades with a distance between the fixation points longer than 1.6⋅ were defined as global saccades (Zangemeister et al., 1995; Vogt and Magnussen, 2007).

The interest in the recognizable subjects in each photograph was operationalized as the total duration of fixation time on the area. To assess the interest in recognizable subjects, the area of each photograph was divided into 16 parts by three horizontal lines and three vertical lines. The four central cells were defined as the central area, and the 12 peripheral cells were defined as the peripheral area. The photographs with recognizable subjects had objects and models in the central area of the whole picture. According to Hypothesis 1, if the interest of novices in recognizable subjects in photographs changed, the rate of viewing time on the central areas would be different in the pre- and post-tests.

## Results and discussion

### Students' eye movements in art appreciation

Descriptive statistics of the eye movement measures included in this study are reported in Table 3. Also, a three-way repeated measures ANOVA [2 (session) × 2 (recognizable subject) × 3 (type of photograph)] was conducted on all the measures individually (see Table 4).

**Table 3:** **Descriptive statistics of eye movement measures**.

		**Educationally intervened photographs**											
		**Control**				**Classic photography**				**Street photography**			
		**Recognizable** **subjects**		**Unrecognizable** **subjects**		**Recognizable** **subjects**		**Unrecognizable** **subjects**		**Recognizable** **subjects**		**Unrecognizable** **subjects**	
**Measures**		**pre**	**post**	**pre**	**post**	**pre**	**post**	**pre**	**post**	**pre**	**post**	**pre**	**post**
Perceptual exploration	Average Fixation Duration (MS)												
	*M*	487.65	450.49	457.14	484.56	515.66	476.48	483.12	440.00	551.92	485.19	449.48	484.93
	*SEM*	11.48	17.75	10.66	19.84	14.10	23.34	23.26	17.70	36.04	25.44	13.44	21.68
Global saccades	Frequency of Global Saccades												
	*M*	14.81	13.46	12.77	11.88	10.35	11.50	12.31	15.73	11.19	12.96	13.54	12.35
	*SEM*	1.01	0.96	1.20	1.06	0.67	1.10	0.74	0.98	0.95	1.03	1.17	1.21
Interest in central area (%)	Viewing Time Ratio on Central Area (%)												
	*M*	44.98	44.28	37.33	42.26	64.73	69.33	48.56	44.66	48.77	46.62	31.26	37.01
	*SEM*	2.98	3.64	2.13	3.51	3.09	3.62	3.96	3.17	3.50	3.92	2.55	2.85

*M, mean*.

*SEM, standard error of the mean*.

**Table 4:** **Results of the three-way ANOVA with repeated measures for eye movement measures**.

**Measures**		***d.f*.**	***MS***	***F***	***p***		**partial**
Perceptual exploration	Average Fixation Duration (M)						
	Session	1	16475.45	2.88	0.12		0.194
	Recognizable subject	1	30632.29	9.96	0.01	^***^	0.454
	Type of photography	2	6946.63	1.28	0.30		0.097
	Session × Recognizable subject	1	28719.70	11.02	0.01	^***^	0.479
	Session × Type of photography	2	4512.93	0.86	0.44		0.07
	Recognizable subject × Type of photography	2	9583.76	1.78	0.19		0.129
	Session × Recognizable subject × Type of photography	2	9407.79	3.05	0.07		0.20
Global saccades	Frequency of Global Saccades						
	Session	1	9.26	0.31	0.59		0.025
	Recognizable subject	1	20.10	1.93	0.19		0.139
	Type of photography	2	9.52	1.08	0.36		0.082
	Session × Recognizable subject	1	0.06	0.00	0.95		0
	Session × Type of photography	2	38.04	4.34	0.03	^*^	0.266
	Recognizable subject × Type of photography	2	78.37	6.32	0.01	^**^	0.35
	Session × Recognizable subject × Type of photography	2	22.94	2.53	0.10		0.174
Interest in central area (%)	The Ratio of Time Viewing Central Area (%)						
	Session	1	79.04	0.31	0.59		0.025
	Recognizable subject	1	6528.73	80.77	0.00	^**^	0.871
	Type of photography	2	4056.22	38.54	0.00	^**^	0.763
	Session × Recognizable subject	1	27.62	0.34	0.57		0.027
	Session × Type of photography	2	11.42	0.13	0.88		0.011
	Recognizable subject × Type of photography	2	793.25	4.61	0.02	^*^	0.28
	Session × Recognizable subject × Type of photography	2	256.69	2.89	0.08		0.194

* *p < 0.05*,

** *p < 0.01*,

*** *p < 0.001*.

**Figure 2:**
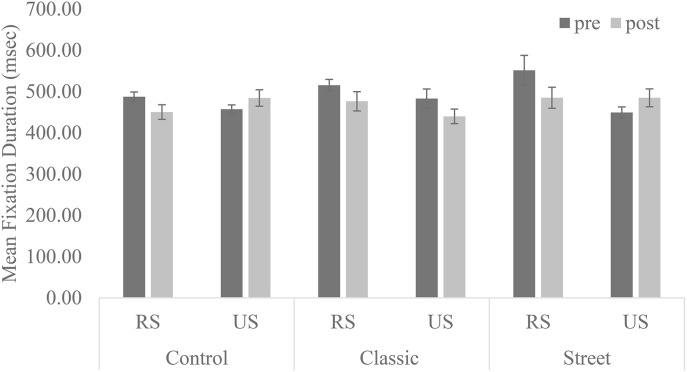
**Perceptual exploration with each type of photography**. RS, Recognizable subjects; US, Unrecognizable subjects. Error bars represent the standard error of the mean.

A change in the appreciation process was found in perceptual exploration and global saccades. The average duration of fixation demonstrated the main effect of a recognizable subject, and was longer in photographs with a recognizable subject than an unrecognizable one [*F*_(1, 12)_ = 9.96, *p* = 0.01, partial η^2^ = 0.45]. The main effect was discernable in the two-way interaction between session and recognizable subject [*F*_(1, 12)_ = 11.02, *p* = 0.01, partial η^2^ = 0.48]. Subsequent analysis showed a simple main effect of session [*F*_(1, 12)_ = 9.24, *p* = 0.01, partial η^2^ = 0.44]. The average duration of fixation decreased from pre-test to post-test with the photographs with a recognizable subject (Figure 2). These results indicate that perceptual exploration became more active with the photographs with a recognizable subject.

**Figure 3:**
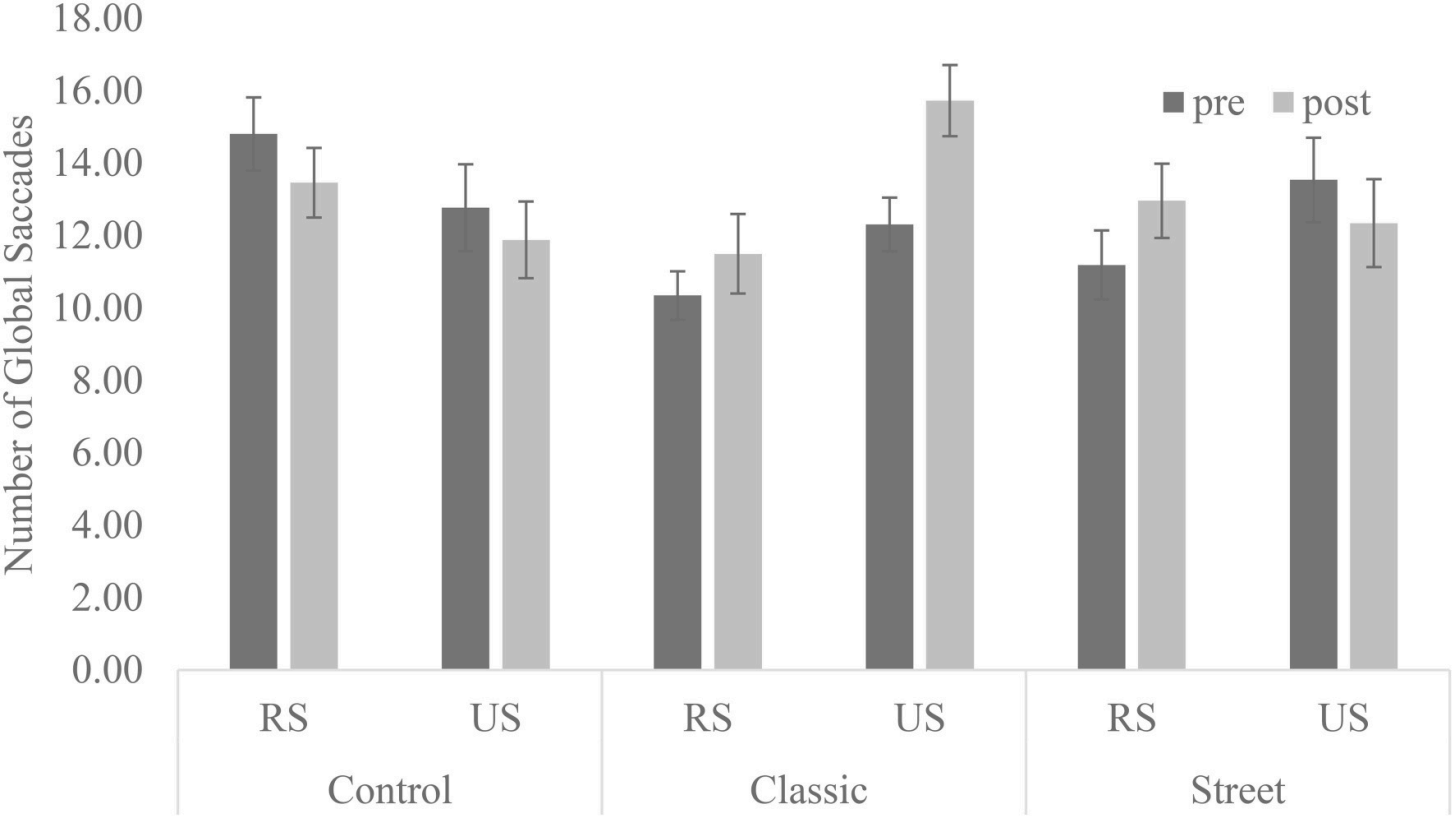
**Frequency of global saccades with each type of photography**. RS, Recognizable subjects; US, Unrecognizable subjects. Error bars represent the standard error of the mean.

Further, the results of global saccades showed a significant interaction between the type of photography across sessions [*F*_(2, 24)_ = 4.34, *p* = 0.03, partial η^2^ = 0.27; Figure 3]. The subsequent analysis showed that the frequency of saccades increased after the course on classic photography [*F*_(1, 12)_ = 3.62, *p* = 0.08, partial η^2^ = 0.23]. The results showed that global saccades increased with the classic photography that was covered in the course.

**Figure 4:**
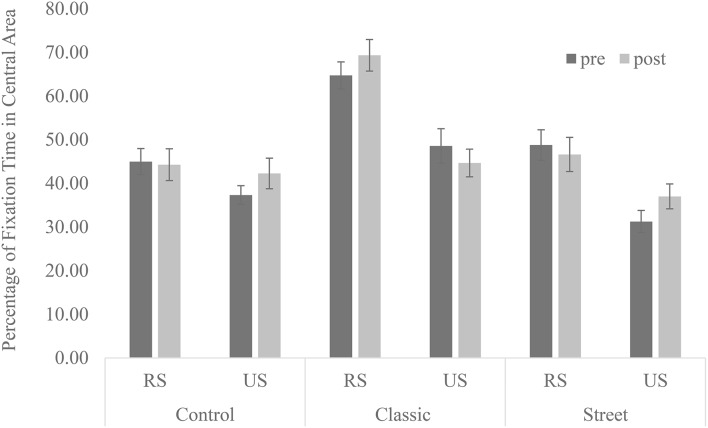
**The interest in central area for each type of photography (%)**. RS, Recognizable subjects; US, Unrecognizable subjects. Error bars represent the standard error of the mean.

However, the interest in recognizable subjects did not change even after the course. The analysis of the ratio of viewing time on the central area showed the main effects of a recognizable subject [*F*_(1, 12)_ = 80.77, *p* = 0.00, partial η^2^ = 0.87] and type of photography [*F*_(2, 24)_ = 38.54, *p* = 0.00, partial η^2^ = 0.76]. Specifically, the rate was higher in photographs with a recognizable subject than an unrecognizable one, and the rate with classic photographs was higher than with other types. Additionally, a two-way interaction effect between subject and type was found [*F*_(2, 24)_ = 4.61, *p* = 0.02, partial η^2^ = 0.28]. The subsequent analysis indicated the simple main effect of a recognizable subject in classic photographs [*F*_(1, 12)_ = 35.75, *p* = 0.00, partial η^2^ = 0.75] and street photographs [*F*_(1, 12)_ = 15.86, *p* = 0.00, partial η^2^ = 0.57]. Specifically, the ratio was higher in photographs with a recognizable subject than without such a subject in both types of photography (Figure 4). Therefore, part of the first and second hypotheses was supported.

### Students' reflections on the effects of the course on their appreciation of art

The interview data after the experiment confirmed that participants had noticed a change in visual information processing in their own photograph appreciation. Of the 13 participants, 11 answered that their way of appreciating art had changed. Also, nine of them explained that they had started to pay attention to the composition and lighting aspects that they had learned about on the course, as described below.

*S1: Before the course, I vaguely viewed people's faces in portraits, and objects in still life. After the course, I started to think about where the light came from and what the form of the object was like, and I imagined the way the light would illuminate it*.

*S2: Previously, I observed the models in the photographs, such as people's faces and buildings, but this time I paid attention to the shadows and lights that I had learned about on the course*.

These interview data indicated that the participants started to appreciate the artistic features by applying the knowledge and techniques they had learned during the course. These verbal protocols provide supporting evidence indicating that the course had helped develop the participants' appreciation process.

## General discussion

The purpose of this study was to examine whether learners changed their viewing strategies in art appreciation after an educational course that included appreciation instruction and training in photo creation, and if so, what kind of changes in viewing strategies there were. The results show that the students' mean duration of fixation decreased when viewing photographs with recognizable subjects, whether or not the photo category was covered in the course. In addition, their global saccades also increased after the course when viewing classic photographs, which were covered in the course. Although, there was no change with the street photographs, which were also covered in the course, the results suggest that it was not possible for the viewing strategies to be transferred to the appreciation of street photographs, and that the change in viewing strategies could not be applied to this specific kind of photographs. However, the students' reflections indicate that they utilized their knowledge of composition and lighting acquired on the course.

### Measuring changes in learners' viewing strategies by eye movement

Although, studies of appreciation in art education have focused on changes or differences in viewers' interpretation using analysis of reports and interview data, no studies to date have examined whether educational interventions can improve learners' viewing strategies in art appreciation. The current study has provided new findings about the change in learners' eye movements after educational interventions, which imply the possibility that eye movements may be suitable measures for examining changes in viewing strategies in art appreciation due to educational interventions. They also indicate that perceptual exploration and global saccades can be used as measures. Hence, it may be suggested that researchers could apply these measures for examining the educational effects on art appreciation in the future.

### Educational methods effective for visual attention in art appreciation

Two kinds of educational methods were included in the course. The first method involved technical training in photo creation. The specific knowledge and techniques for photo creation in the course helped the students to visualize the creation process of photos during art appreciation. The second method involved dialogic appreciation and imitation of fine art photography with fellow students and an expert artist. The expert artist provided interpretations of art historical knowledge and techniques used in the artwork. In addition, imitation of artwork provided opportunities for the students to practice creating artwork. These challenges may have helped the students to acquire procedural knowledge and techniques for creation, and they may also have made the knowledge and techniques more applicable, not just to creation but also to appreciation.

It is interesting that the change in the students' viewing strategies did not appear with all the types of photography. The educational course in this study, the “Artistic Creation” course, covered classic and street photographs. There was a significant change in viewing photos in a category not covered in the course. Fixation times decreased for photos with recognizable subjects in all categories. By contrast, with the types of photography that had been covered in the course, the change in viewing strategies in photo appreciation was not universal. Appreciation of classic photographs changed after the educational intervention, whereas no changes were found with street photographs. What could be inferred from the results? One possible way to interpret this difference in that the change in viewing strategy relates to the different creation processes between classic and street photographs. The educational intervention that focused on the method of approaching the models as a creation process for street photographs focused on the camera technique for classic photographs. Thus, it could be suggested that the students visualized how to approach the models rather than explore specific camera techniques when viewing street photographs; however, the interpretation process may not be reflected in the eye movements. The results of the interviews provided supporting evidence, indicating that students applied their knowledge of camera techniques, such as composition and lighting, but did not describe their knowledge of how to approach the models.

The other possibility relates to the generalizability of knowledge and experience acquired through the educational intervention. If the knowledge was more generally applicable, the change in appreciation might be seen with the other types of artwork. Alternatively, if the students spent more time developing their knowledge, they may have applied it to other types of artwork. If it can be concluded from the results of the interview data that the students utilized the knowledge of composition and lighting, it might be argued that they could have applied the knowledge not only to classic photographs but also to street and control photos, still life and crowds, which also contain composition and lighting components. Why were such results not acquired? We can assume that the students did not apply their knowledge in viewing photographs other than classic ones because their knowledge application was limited to certain kinds of photographs. Future studies are required to examine how knowledge is acquired in educational intervention work in art appreciation.

### Limitations and future work

Further examinations are required on the validity and reliability of the individuals' change in eye movement in art appreciation. As a first step, researchers should examine what causes the change. If they suspect that there is an educational effect, they should verify whether there is an educational effect or not by controlling the educational intervention in different aspects. In particular, researchers should study both learners who participate in a course and a control group comprised of individuals who do not participate in a course. Also, it would be meaningful to examine whether the contents of educational interventions are effective or not by designing a variety of interventions. For instance, it would be significant to compare differences in effect between instructions in technical knowledge and art history. Furthermore, researchers should examine how the characteristics of photographs viewed in experiments affect the change in learners' viewing strategies, because it may be inferred that familiarity, liking, and interest influence art viewing (Brieber et al., 2014). If these examinations provide findings about robust methods to measure learners' development of appreciation, it may become possible to examine the educational effect of art courses by behavioral measures, such as eye movements.

It is also significant to study how changes in the eye movements of learners affect their interpretation of artworks in art appreciation. For instance, a change in viewing strategies may influence the amount and quality of the information that can be used by viewers in their interpretation. It may therefore be assumed that an educational course can improve the interpretation of artwork, as well.

Additionally, it cannot be denied that the participants may have controlled their eye movements after the explanation of the study purpose, even though they were not informed about the hypotheses. To avoid this, researchers might provide other information about the study being conducted in order to divert the participants' attention from their own eye movements, providing both a distraction and a debrief.

## Conclusion

A reliable method to measure the appreciation process is required for the evaluation of educational effects. As an initial step in this direction, this study examines whether the processes of appreciation in people untrained in art change after educational interventions, and if so, what kinds of changes can be observed. An artistic photo creation course was conducted to provide students with technical and procedural knowledge to promote their art appreciation. Additionally, the students participated in quasi-experiments to measure their eye movements before and after the course. The results suggest that students' viewing strategies changed following the course when viewing classic photos that had been discussed on the course. In addition, the students' feedback after the course demonstrated their improved awareness of technical and procedural knowledge, which they applied in their appreciation.

The results of this study suggest the possibility that eye movements may function as measures for examining changes in viewing strategies in art appreciation due to educational interventions. Specifically, the possible measures are perceptual exploration and global saccades. The findings of this study are important for the field of art education, as they suggest that behavioral measures may change in individuals after educational interventions, and that this change is measurable by eye movement.

## Author contributions

CI, KY, and TO contributed to the conception and design of this study, the analysis and interpretation of data, drafting and revising the study, including the final approval of the version to be published, and accepted the agreement to be accountable for all aspects of this paper. The data collection was made by CI.

### Conflict of interest statement

The authors declare that the research was conducted in the absence of any commercial or financial relationships that could be construed as a potential conflict of interest.
